# Magnesium Balance in Chronic Kidney Disease: Mineral Metabolism, Immunosuppressive Therapies and Sodium-Glucose Cotransporter 2 Inhibitors

**DOI:** 10.3390/ijms26125657

**Published:** 2025-06-13

**Authors:** Juan Miguel Díaz-Tocados, Maria Jesús Lloret, Juan Diego Domínguez-Coral, Adria Patricia Tinoco Aranda, Leonor Fayos de Arizón, Elisabet Massó Jiménez, Jordi Bover, José Manuel Valdivielso, María Encarnación Rodríguez-Ortiz

**Affiliations:** 1 Vascular and Renal Translational Research Group, Biomedical Research Institute of Lleida (IRBLleida), 25198 Lleida, Spain; jdominguez@irblleida.cat (J.D.D.-C.); josemanuel.valdivielso@udl.cat (J.M.V.); 2 Cooperative Research Network Oriented Towards Health Outcomes (RICORS), Carlos III Health Institute (ISCIII), 28029 Madrid, Spain; emassoj.germanstrias@gencat.cat (E.M.J.); jbover.ics@gencat.cat (J.B.); marien_rguez@hotmail.com (M.E.R.-O.); 3 Nephrology Department, Fundació Puigvert, 08025 Barcelona, Spain; mjlloretcora@gmail.com (M.J.L.); atinocomd@gmail.com (A.P.T.A.); l.fayosarizon@gmail.com (L.F.d.A.); 4 Institut de Recerca Sant Pau (IR Sant Pau), Departament de Medicina, Universitat Autònoma de Barcelona, 08041 Barcelona, Spain; 5 Nephrology Department, Germans Trias i Pujol University Hospital, REMAR-IGTP Group, 08916 Badalona, Spain; 6 Nephrology and Cell Damage in Chronic Inflammation Research Group, Nephrology Department, Reina Sofia University Hospital, Maimonides Institute for Research in Biomedicine of Cordoba (IMIBIC), University of Cordoba, 14004 Cordoba, Spain

**Keywords:** magnesium, chronic kidney disease, CKD-MBD, renal transplant patients, SLGT2 inhibitors

## Abstract

It is now widely recognized that maintaining magnesium (Mg) homeostasis is critical for health, especially in the context of chronic kidney disease (CKD). Patients with CKD commonly develop hyperphosphatemia and secondary hyperparathyroidism, which are controlled by therapies targeting intestinal phosphate absorption and circulating calcium levels or by modulating parathyroid calcium sensing. Notably, Mg supplementation may provide dual benefits by promoting bone formation and maintaining normal mineralization with slightly elevated serum levels. Importantly, low Mg levels are associated with mortality risk in CKD, highlighting the importance of maintaining adequate serum Mg levels in these patients. Particularly, kidney transplant (KT) patients have lower circulating Mg levels, likely due to interactions with immunosuppressive treatments. Sodium-glucose co-transporter 2 (SGLT2) inhibitors have shown survival benefits in CKD and increased serum Mg levels, suggesting that Mg regulation may contribute to these outcomes. Overall, Mg plays a key role in CKD-associated mineral and bone disorders (CKD-MBD). Thus, understanding the mechanisms underlying the alteration of Mg homeostasis in CKD could improve clinical outcomes. This review summarizes the basic and clinical studies demonstrating (1) the key actions of Mg in CKD-MBD, including secondary hyperparathyroidism and bone abnormalities; (2) the distinctive profile of KT patients for Mg homeostasis; and (3) the interaction between commonly used drugs, such as SGLT2 inhibitors or immunosuppressive treatments, and Mg metabolism, providing a broad understanding of both the key role of Mg in the context of CKD and the treatments that should be considered to manage Mg levels in CKD patients.

## 1. Introduction

In recent decades, magnesium (Mg) has transitioned from being considered a cation of minor interest for health to an important factor involved in multiple key functions essential for systemic metabolism and survival. In the context of chronic kidney disease (CKD), numerous basic and clinical research studies have demonstrated the key role of Mg in the prevention of multiple complications. Notably, both intra- and extra-cellular Mg levels can regulate the activity of several signaling pathways involving key receptors and transporters, such as the Transient Receptor Potential Cation Channel Subfamily M Member 7 (TRPM7) [1], Calcium-Sensing Receptor (CaSR) [2], N-methyl-D-aspartate (NMDA) receptor [3], P2X purinoceptor 7 (P2X7) receptor [4], and Magnesium Transporter 1 (Magt1) [5], among others. Since these receptors are widely expressed in numerous cell types, alterations in Mg levels due to metabolic or nutritional changes can compromise multiple functions at the systemic level, such as inflammation [6], bone and mineral metabolism [7], myogenic differentiation [8], endothelial function, and oxidative stress [9]. Conditions leading to alterations in Mg levels [10], particularly hypomagnesemia, contribute to the development of mineral and bone disorders [11], cardiovascular complications [12,13,14], sarcopenia [15], and erythrocyte disorders and anemia [16]. Notably, these complications generally occur with renal insufficiency; thus, maintaining an adequate Mg balance in patients with CKD should be beneficial at multiple levels. Treatments such as immunosuppressive drugs, which are administered to kidney transplant (KT) patients, or sodium-glucose co-transporter 2 (SGLT2) inhibitors, initially used to treat diabetes, can significantly contribute to Mg homeostasis, at least at the circulating levels [17]. It is important to highlight that low Mg levels are associated not only with increased cardiovascular mortality but also with all-cause mortality in patients with CKD [18,19,20], highlighting the potential importance of maintaining adequate Mg levels. In patients with CKD treated with hemodialysis, lower levels of serum Mg are associated with a higher risk of vascular calcification (VC) and increased fracture risk [21]. Numerous mechanisms have been proposed for the actions of Mg in the inhibition of VC, including the prevention of pathological calciprotein particle (CPP) development, interference in hydroxyapatite formation, and counteracting phosphate-induced calcification by inhibiting pathways involved in osteogenic vascular smooth muscle cell transdifferentiation, such as Wnt/β-catenin or Bone Morphogenic Protein-2 (BMP2) signaling pathways [22,23,24,25]. Despite numerous studies supporting the preventive effect of Mg on the development of VC, the results of clinical trials are not consistent [26,27]. Although this topic is of high relevance, the effects of Mg on VC in the CKD context are not extensively discussed in this review, as a detailed evaluation of this issue has already been covered by Zaslow S.J., et al., [28] in the first volume of this Special Issue.

In this review, we highlight the crucial role of Mg in the regulation of bone and mineral homeostasis in patients with CKD, which is related to numerous CKD-associated complications, as well as the relevant interactions among drugs that are commonly used in renal patients and Mg balance, such as immunosuppressive therapies in KT patients and SGLT2 inhibitors. The objective of this work is to provide concise knowledge on the principal actions of Mg in the context of CKD and mineral metabolism, as well as the potential effects of common treatments on the alteration of Mg homeostasis.

## 2. Magnesium and CKD-MBD

### 2.1. Magnesium and Secondary Hyperparathyroidism

#### 2.1.1. Magnesium and Parathyroid Function

Adequate production of parathyroid hormone (PTH) is essential for maintining mineral homeostasis. The presence of CaSR on their surface allows parathyroid cells to sense small changes in the concentration of extracellular Ca and respond with appropriate PTH secretion [29]. The binding of Ca to CaSR triggers a signaling cascade that ultimately leads to the activation of the phospholipase A2-arachydonic acid-mitogen-activated protein kinase (PLA2-AA-MAPK) pathway [30,31]. The activation of this cascade not only modulates PTH synthesis but also the expression of CaSR, the Vitamin D Receptor (VDR), Klotho, and the Fibroblast Growth Factor Receptor 1 (FGFR1) [32,33,34,35], all of which are crucial elements for parathyroid function.

Although Ca is the main agonist for CaSR, other divalent cations, such as Mg, may also activate the receptor and should be considered in the management of secondary hyperparathyroidism (SHPT). Several studies have suggested a relationship between changes in Mg and PTH secretion. In humans, elevated Mg levels are associated with significant reductions in PTH levels [36,37]. These findings were further confirmed in experimental studies carried out in vitro [38,39,40], which reported that the inhibitory effect of Mg requires the presence of moderately low Ca in the extracellular medium. Furthermore, in addition to modulating PTH levels, Mg also increases the expression of CaSR, VDR, FGFR1, and Klotho, which represent additional mechanisms of inhibition of parathyroid function [41].

#### 2.1.2. Magnesium and Clinical Management of Secondary Hyperparathyroidism

Secondary hyperparathyroidism, characterized by excessive PTH synthesis and secretion, along with parathyroid cell hyperplasia, develops as CKD progresses. Secondary hyperparathyroidism begins as an adaptive response but becomes maladaptive as kidney function declines, leading to biochemical disturbances and increased morbidity and mortality [42]. The modulatory effect of Mg on PTH secretion has also been established in the context of renal dysfunction [43,44,45,46]. Hence, the previously described evidence may lead to the hypothesis that the therapeutic administration of Mg may potentially impact the progression of SHPT.

Phosphate (P) binders are used in clinical practice to manage hyperphosphatemia. Aluminum- and Ca-based P binders were frequently used in the past, but these molecules have been substituted with newer drugs due to their toxicity and the risk of Ca overload, respectively. Mg-containing P binders are among these new compounds. In the 1980s, the use of Mg hydroxide in combination with oral Ca carbonate was proven to be effective in suppressing PTH in patients undergoing renal replacement therapy, and it also increased circulating Mg concentration [47,48]. In a very interesting study, O’Donovan et al. [49] found that the administration of Mg carbonate after discontinuing therapy with Mg hydroxide produced a marked suppression of PTH in 12 out of the twenty-eight patients treated with HD included in the study while maintaining stable levels of Ca and P, thus controlling SHPT. Spiegel et al., [50] compared the efficacy of Mg carbonate alone versus Ca acetate in a prospective, randomized, open-label trial involving thirty patients treated with HD. Both compounds were equally effective in controlling P levels, and serum Mg levels increased in the Mg-treated arm according to Mg intake. However, no changes were observed in PTH levels, which could be explained by the small sample size and short duration of the study. The effectiveness of oral Mg carbonate (3 to 9 tablets/day; 250 mg/tablet) was also compared with that of oral Ca carbonate (3 to 9 tablets/day; 420 mg/tablet) in a single-center randomized controlled clinical trial. After 6 months, both compounds were equally effective, and the number of patients in both arms with CaxP product, P, and PTH levels within the ranges recommended by the K/DOQI guidelines was similar. However, patients treated with Mg carbonate had lower serum Ca levels and did not experience hypercalcemic episodes [51]. Several studies have assessed the efficacy of Mg-based compounds in combination with Ca-containing P binders, allowing for a reduction in the Ca load. Delmez et al., [52] compared the combination of Mg and Ca carbonate versus Ca carbonate alone in patients treated with HD, finding similar results in the control of SHPT but with considerably lower Ca intake in the group receiving the combined therapy. In the long term, this combination was effective in managing SHPT and, remarkably, also in preventing the progression of vascular calcification, which might be related to the control of hyperphosphatemia [53]. The CALMAG Study assessed the efficacy of the combination of Mg carbonate and Ca acetate in comparison with sevelamer-HCl in patients undergoing HD. The combination of Mg carbonate/Ca acetate effectively controlled SHPT, inducing a greater decrease in phosphorus and PTH levels than sevelamer-HCl [54]. Although the results derived from the previous studies are apparently quite robust, several limitations should be acknowledged, such as the small sample size [47,48,49,50], which might have prevented the authors from finding differences in the effects on blood pressure, serum bicarbonate, or gastrointestinal side effects. In addition, other studies did not report any bone effects derived from the administration of Mg-based molecules [51,54]. Despite these positive results in terms of SHPT management, it must be acknowledged that, in clinical settings, it may be difficult to separate the PTH-lowering effect induced directly by Mg from that caused by the reduction in circulating P levels [55]. In this regard, McGonigle et al. [56] specifically assessed the influence of Mg on circulating PTH in patients undergoing stable HD by increasing the concentration of Mg in the dialysate and found a marked and significant decrease in serum PTH levels after 10 weeks, irrespective of changes in serum Ca, P, or vitamin D concentrations. A different approach was used in a prospective study carried out one decade later. In this work, patients with CKD treated with peritoneal dialysis were subjected to Mg depletion using Mg-free dialysate. Under these conditions, a significant increase in PTH levels was observed at both weeks 4 and 8, with no changes in Ca and P [57]. Although the sample size was small in this study, the reduction in Mg levels was also confirmed at the intracellular level. Altogether, these findings support the notion that Mg directly modulates parathyroid function also in the setting of SHPT, although other indirect mechanisms cannot be ruled out.

Finally, it is worth mentioning that the relationship between Mg and PTH is bidirectional. While high Mg levels inhibit PTH secretion, PTH also influences the Mg status. This relationship was elegantly demonstrated in patients with CKD by Fang et al. [58], showing that parathyroidectomy was followed by a marked and significant drop in serum Mg, as well as calcium and phosphate, which should be a result of the hungry bone syndrome since all these elements are part of the bone mineral composition [59]. Thus, it may be reasonable to think that the elevations in PTH levels in SHPT also contribute to the derangements in Mg metabolism observed in patients with CKD.

#### 2.1.3. Risk of Hypermagnesemia

Despite the potential benefits of Mg administration in CKD, its regular use raises concern due to the risk of hypermagnesemia as a consequence of impaired renal clearance [60]. Nevertheless, although no clinical effects have been observed with levels of Mg up to 3.65 mg/dL [61], monitoring Mg status may be advisable. The possible bone effects associated with the therapeutic use of Mg constitute another reason for concern [62]. Conversely, recent studies have associated Mg intake with higher bone mineral density [63,64], as well as with elevated levels of markers of bone formation [65].

In summary, the evidence suggests that the administration of Mg may have a potential impact on the clinical management of SHPT, either directly by modulating PTH production and release or indirectly by acting as a negative regulator of parathyroid function and reducing circulating P. However, monitoring Mg status might be considered to avoid potential adverse effects related to the development of hypermagnesemia, such as laxation, hypotension, nausea, or vomiting [66,67].

### 2.2. Magnesium and Bone Health

#### Magnesium Actions on Osteogenesis and Mineralization

Maintaining adequate magnesium (Mg) levels is essential for bone health. Notably, over 60% of the total body Mg is stored in the bone, primarily within the mineralized matrix of the cortical and trabecular compartments. These sites serve as dynamic reservoirs that help regulate physiological plasma Mg concentrations [68]. Moreover, Mg can directly regulate bone homeostasis by acting on both bone cells and the matrix or indirectly by regulating multiple hormones and functions that control mineral homeostasis.

In the general population, the recommended cut-off for hypomagnesemia is 0.85 mM, while the proposed normal serum Mg reference range is 0.85–0.95 mM [69]. A strong correlation between serum Mg and bone Mg content has been reported. It has been suggested that bone Mg content in patients with CKD is higher than that in control subjects with normal renal function. In this regard, one-third of the total bone Mg is deposited on the bone surface and is easily exchangeable, while the proportion of Mg embedded within the bone must undergo resorptive processes to be released into circulation [70]. However, groups in this study had a small sample size (*n* < 10 per group), and bone samples were obtained from cadavers. Nevertheless, although mild hypermagnesemia (between 1.05 and 2.2 mM) is generally considered asymptomatic [71], some studies have reported that high Mg levels may lead to defective mineralization, at least in part due to an increase in whitlockite formation, a component of the bone matrix with higher solubility than hydroxyapatite. Technically, Mg concentrations up to 3 mM should not promote inorganic calcium-deficient hydroxyapatite formation [72]. In fact, in an in vitro study using distinct proportions of hydroxyapatite and whitlockite, the authors observed that adding whitlockite increased osteogenic differentiation of mesenchymal stem cells after 14 days, showing a peak when 25% whitlockite was added [73]. Another study has also shown that whitlockite has higher osteogenic potential than hydroxyapatite and that increasing Mg concentration up to 5mM resulted in higher mineralization and alkaline phosphatase activity [74]. These effects were related to soluble Mg ions released from whitlockite and intracellular signaling activation, as Magt1 inhibition abolished the Mg pro-osteogenic actions. On the other hand, Zhang J. et al. [75] reported that Mg supplementation at 0.25 mM (reaching a final concentration of 1.06 mM, considering the MgSO_4_ in the culture media formulation) increased mineralization of mouse bone marrow mesenchymal stem cells under osteogenic differentiation; however, they observed a dose-response inhibitory effect of Mg supplementation (reaching a final concentration of ~1.3 mM). Similarly, they observed that growing mice fed a high Mg diet showed lower bone mineralization than those on a normal Mg diet. The absolute values in the in vitro study slightly differ from those in other studies reporting that Mg concentrations up to 1.8 mM, which are values within the asymptomatic range of mild hypermagnesemia, produce increased osteogenesis and mineralization during bone marrow mesenchymal stem cell differentiation [76].

In mice, the administration of ionized Mg at early stages of bone injury accelerates bone healing, increasing bone volume by promoting osteoblast activity while decreasing osteoclast activity [77]. The authors observed that delayed or sustained administration of ionized Mg after bone injury did not produce significant effects on bone volume and bone cell activity. Notably, the authors observed indirect osteo-anabolic and anti-resorptive actions of ionized Mg, which were induced by TRPM7-mediated regulation of gene expression in bone marrow monocytes/macrophages, leading to immunomodulation of the bone microenvironment (downregulation of TNFα, IL-1β, and IL-10 and upregulation of IL-8, among others). In vitro, conditioned medium from Mg-treated macrophages (up to 8 mM MgCl_2_) or IL-8 enhanced osteogenic differentiation and mineralization of mesenchymal stem cells in a dose-dependent manner, indicating a direct connection between macrophages and bone marrow mesenchymal stem cells in the proper regulation of osteogenic differentiation, which is enhanced by high Mg levels. In animal models of CKD, dietary Mg supplementation to achieve sufficient serum Mg levels to prevent vascular calcification appeared to be safe for bone mineralization. [78].

Therefore, while extremely excessive Mg seems to be detrimental to bone mineralization, it has been demonstrated that osteogenic differentiation and collagen production are increased by slightly high Mg levels, promoting in vivo bone mineralization/formation. However, determining the optimal range for the positive effects of mild hypermagnesemia is key to translating these bone benefits into clinical practice.

### 2.3. Magnesium and Bone Homeostasis

Since Mg is embedded in the bone microstructure, specifically in the hydroxyapatite crystals, it acts directly on the bone matrix properties, contributing to bone strength and flexibility while also orchestrating bone cell activity. Animal studies have shown that hypomagnesemia negatively affects bone formation by reducing osteoblast activity. Of note, it has been observed that the levels of alkaline phosphatase and osteocalcin, both markers of osteoblast function, are reduced in conditions of hypomagnesemia [79]. Similarly, hypomagnesemia can lead to increased bone resorption and decreased bone density through its effects on osteoclasts [63].

Indirectly, Mg regulates bone metabolism through its effects on parathyroid hormone (PTH) and vitamin D. In many species, low Mg levels hinder the secretion of PTH and make target organs less responsive. Since PTH signaling involves increasing cyclic AMP through the activation of adenylate cyclase, an enzyme that requires Mg as a cofactor, this resistance to PTH may be partly due to the reduced activity of adenylate cyclase. Furthermore, 25-hydroxycholecalciferol-1-hydroxylase depends on Mg; thus, a deficiency in Mg could lead to a decrease in enzyme activity and low serum calcitriol concentrations [80].

The involvement of altered Mg homeostasis in bone disorders has been documented in many studies. Mederle et al., [81] conducted a study in which they analyzed the relationship between bone mineral density (BMD) and the serum levels of several bone turnover markers and Mg in postmenopausal women with osteoporosis (*n* = 132) compared to healthy postmenopausal women (*n* = 81), noticing that patients with osteoporosis had significantly lower serum Mg levels. Indeed, levels of Mg correlated positively with BMD. Despite the promising results in favor of the role of hypomagnesemia in osteoporosis, the distinct ages between groups and the limited number of circulating biomarkers of bone cell activity, bone-specific alkaline phosphatase (BALP) for bone formation, and Tartrate-Resistant Acid Phosphatase Isoform 5B (TRAP5B) for bone resorption are important limitations of this clinical study. Nevertheless, similar results were found in a meta-analysis of seven case-control studies involving 1349 postmenopausal women, where the pooled analysis indicated that osteoporotic women had lower serum Mg levels than healthy controls [82].

Likewise, several studies have linked hypomagnesemia with an increased risk of fracture [83]. An observational cohort study conducted in Finland investigated the relationship between serum Mg concentration and fracture risk in adult men, finding that the relative fracture risk was higher in the group with lower Mg levels (HR: 1.8; 95% CI:1.1–2.94) [84]; however, despite the relevant sample size (2245 participants included), the analysis did not adjust for important confounding factors, such as Vitamin D levels, BMD, and the presence of prevalent conditions such as inflammatory diseases, which would strongly influence the results. The effects of oral Mg supplementation (15 mmol/24 h) on the regulation of bone cell activity have also been demonstrated in a small cohort of young men compared to a control group (*n* = 12 per group) [85]. The authors observed that Mg supplementation resulted in decreased levels of serum markers of bone formation and resorption, which was associated with a concomitant reduction in serum PTH levels in the Mg group. However, this study showed inconsistencies between Mg intake and serum Mg^2+^ levels.

The relationship between Mg and fracture risk in patients treated with HD has also been investigated. The incidence of hip fractures was significantly higher in patients with low Mg levels. Patients with serum Mg levels below 2.3 mg/dL had a 23% higher fracture risk than those with Mg levels above 2.9 mg/dL. Thus, mild hypermagnesemia (above 2.9 mg/dL) is associated with a lower risk of hip fractures in patients treated with HD [86].

Although bone changes have been shown to be associated with Mg deficiency, it is estimated that up to 10% of the general population does not consume adequate amounts of this ion. The question that arises is whether Mg supplementation is beneficial. The impact of increased dietary Mg intake on the treatment and prevention of osteoporosis has yielded conflicting results. Prospective studies have investigated the effects of oral Mg supplementation (Mg citrate, 1830 mg/day) on biochemical markers of mineral metabolism in 10 postmenopausal women compared with placebo (*n* = 10), showing a significant reduction in PTH and increased levels of osteocalcin (a hormone produced by osteoblasts during bone formation) in the Mg group, suggesting increased osteoblast differentiation despite the reduction in PTH levels [87]. Thus, a decrease in bone resorption, together with an increase in bone formation, could increase bone volume and mineralization. Similarly, a randomized, double-blind, placebo-controlled study enrolling healthy adolescent female subjects with dietary Mg intake lower than 220 mg/day (*n* = 120) observed that oral Mg oxide supplementation (300 mg/day) significantly increased bone mineral content at the hip site after 12 months of treatment [88], suggesting a positive effect of Mg supplementation on net bone formation.

Studies on supplementation via dialysate have also been carried out in patients treated with HD, demonstrating that increased Mg concentration in the dialysis fluids resulted in higher serum Mg levels, which were inversely associated with serum PTH levels [89]. In addition, in a post-hoc analysis, it was shown that increased Mg concentration from 1.0 mEq/L (0.5 mM) to 2.0 mEq/L (1 mM) in the dialysate fluid for 4 weeks (*n* = 28) resulted in higher levels of serum BALP and decreased TRAP5b in comparison with patients receiving commonly used dialysate Mg (1.0 mEq/L; *n* = 29), indicating increased bone formation and decreased resorption, despite the lower serum PTH levels observed [90]. The authors also observed a reduction in serum inflammatory markers in patients on high dialysate Mg, which was associated with the prevention of secondary (pathological) CPP formation. However, this analysis was not adjusted for potential confounding factors that could have contributed to the observed effects.

**Figure 1:**
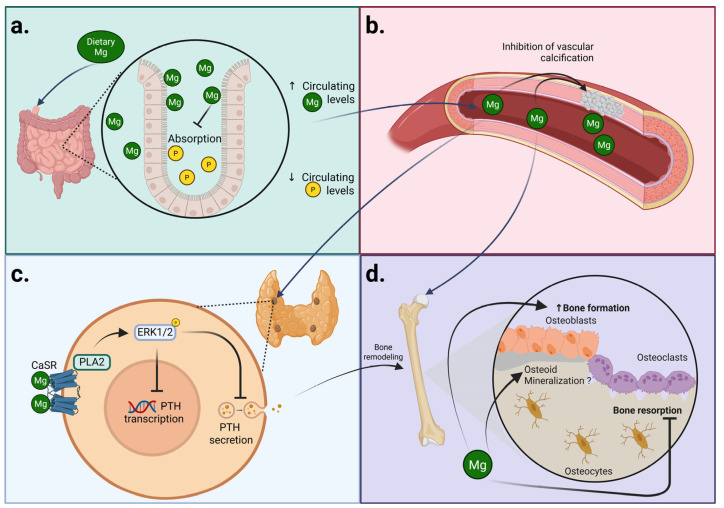
Magnesium effects on bone and mineral metabolism in the CKD context disease (**a**) Magnesium (Mg) supplementation acts as a “phosphate binder,” decreasing phosphate uptake in the gut while increasing circulating Mg levels. (**b**) At the vascular level, circulating Mg inhibits vascular calcification, thus preventing cardiovascular disease. (**c**) Ionized Mg activates the Calcium-Sensing Receptor (CaSR) in parathyroid cells and inhibits parathyroid hormone (PTH) production and secretion. (**d**) The Mg-induced PTH decrease could, in part, reduce bone remodeling; however, Mg exerts direct bone effects by increasing osteoblast activity and inhibiting osteoclastic action, while the Mg effects on mineralization may depend on the Mg range, with a probable deleterious effect if Mg levels are too high. Created in BioRender. Domínguez Coral, J. (2025) https://BioRender.com/biw1hmi.

Whether these observations translate into a lower risk of fractures is still unknown, although a small retrospective study in patients treated with HD found that serum Mg levels below 1.03 mmol/L were associated with a 2.3-fold increased risk of fracture in comparison with higher serum Mg levels. A graphical summary of the effects of Mg on CKD-MBD is depicted in Figure 1.

## 3. Magnesium in Renal Transplant Patients

Hypomagnesemia is frequently observed after KT, particularly within the early post-transplant period, extending from the initial weeks to the first year [91]. This effect is largely attributed to the use of calcineurin inhibitors (CNIs), which induce renal Mg wasting. Hypomagnesemia has been shown with tacrolimus in short- and long-term (1 year) cross-sectional studies with relatively small sample sizes [92,93] and in multicenter clinical trials [94]. Van de Cauter et al., observed that ~20% of KT patients showed persistent hypomagnesemia for at least 5 years [95]. This is important in clinical practice since hypomagnesemia has been linked to new-onset diabetes in transplant patients [96]. Moreover, numerous studies have reported an association between low serum Mg levels and increased risk of all-cause mortality [97,98,99,100], which could be related, at least in part, to the effects of Mg on cardiovascular protection.

The pathophysiology of post-KT hypomagnesemia has been well established in several preclinical studies in animal models, with increased renal Mg wasting as a central mechanism. Thus, rats treated with calcineurin inhibitors, such as tacrolimus, show impaired renal tubular Mg reabsorption through the downregulation of Transient Receptor Potential Cation Channel Subfamily M Member 6 (TRPM6) expression in the distal convoluted tubule [101]. Similarly, mammalian target of rapamycin (mTOR) inhibitors also produce hypomagnesemia in rats [102], which would be mediated, at least partly, by inhibition of epidermal growth factor (EGF)-induced regulation of TRPM6 expression, as demonstrated by in vitro studies in renal tubular epithelial cells [103]. In addition, animal studies have shown that rapamycin may also induce hypomagnesemia through inhibition of the Sodium-Potassium-2 Chloride co-transporter 2 (NKCC2) expression in the thick ascending limb, an action that could be prevented by administration of the antidiabetic drug Rosiglitazone [104].

In a retrospective study of 138 participants, switching from CNIs to mTOR inhibitors was associated with an increase in Mg levels [105]. Of note, a recent preclinical study in rats demonstrated that voclosporin, a new-generation CNI, does not modify urinary Ca excretion or serum Mg levels in comparison with tacrolimus because voclosporin apparently does not affect the expression of TRPM6 and other Mg transporters at the renal distal tubule [106]. In addition, this study observed that tacrolimus reduced urinary EGF, while voclosporin did not, suggesting that voclosporin may exert a potential advantage in slowing CKD progression, as urinary EGF levels have been indirectly associated with the decline in renal function [107].

However, CNIs are not the only contributors to post-KT hypomagnesemia. The immediate post-transplant period represents a “breeding ground” for electrolyte imbalances, with multiple factors contributing to Mg depletion, including potential acute changes in renal function [93], changes in volume expansion [108], metabolic acidosis [109], severe diarrhea [110], and other concomitant medications such as proton pump inhibitors (PPIs), which are commonly prescribed in KT patients with an elevated gastrointestinal pH [111,112]. Additionally, PPIs increase transepithelial resistance by downregulating the TRPM6 channel responsible for Mg absorption [113]. This disruption is particularly problematic with either high PPI doses, prolonged use, or concurrent diuretic therapy, situations that are frequently observed in KT patients.

Beyond its identification as a common post-transplant feature, understanding the repercussions of hypomagnesemia in KT recipients is key to preventing its associated complications. While patients with KT may suffer diverse and nonspecific clinical symptoms that can significantly impact their quality of life, particularly during a period of drastic changes in their medication regimen and exposure to various adverse effects, there are also more subtle consequences that may affect graft viability and global mortality in the long term. The relationship between serum Mg levels and graft function remains unclear due to the limited available data. However, the association between hypomagnesemia and graft function and survival is controversial, with clinical studies suggesting both beneficial and detrimental effects. On the one hand, hypomagnesemia has been implicated in mechanisms contributing to delayed graft, and retrospective studies have demonstrated a greater decline in allograft function in patients with lower Mg levels compared to those with normal levels [114]. On the other hand, another study observed that KT patients with lower serum Mg levels showed longer graft survival [115]. Despite the controversial results in KT patient studies, preclinical studies in cyclosporine-treated rats have demonstrated a protective effect of Mg on the development of tubular atrophy and interstitial fibrosis [116]; however, more clinical trials in KT patients are needed to clarify whether magnesium homeostasis is directly involved in graft survival.

Furthermore, post-transplant diabetes mellitus (PTDM) significantly contributes to morbidity and mortality after solid organ transplantation, highlighting the importance of identifying possible, modifiable risk factors [117]. In this sense, hypomagnesemia has emerged as an independent risk factor for PTDM, although there is some controversy. Its diabetogenic effects are not yet well understood, but it has been hypothesized to involve modulation of insulin action and sensitivity via tyrosine kinase alterations at the insulin receptor level, as well as the induction of inflammation and oxidative stress [118,119]. Van Laecke et al., [96] first identified serum Mg as an independent predictor of PTDM in a cohort of 254 KT patients (179 non-PTDM patients and 75 PTDM patients), even after adjusting for classical risk factors and CNI use. Similarly, Huang et al. [120] confirmed this relationship in a retrospective cohort of 948 non-diabetic KT recipients. A prospective study of 167 KT recipients found that a lower serum Mg trajectory within the first year was an independent risk factor for developing PTDM, with a prevalence of 17% after one year [121].

In this respect, Mg supplementation may offer a safe strategy to mitigate these risks; however, consensus is lacking due to the limited intestinal absorption of conventional formulations. Novel formulations, such as Sucrosomial Mg, have shown promising results in an interventional study enrolling a small cohort of KT patients [122]. In order to prevent hypomagnesemia, SGLT2 inhibitors may represent a game-changing approach in this population, given their ability to considerably increase plasma Mg levels. This topic is further discussed in the following section.

## 4. Magnesium and SGLT2 Inhibitors

Hypomagnesemia is a relatively common electrolyte disorder, occurring in 2–15% of the general population, depending on the selected lower limit of serum Mg levels. Moreover, some conditions, such as alcoholism, type 2 diabetes mellitus (T2DM), and patients taking certain drugs, such as PPIs, diuretics, antibiotics, and immunosuppressive drugs, may contribute to the development of hypomagnesemia [68]. In individuals with T2DM, hypomagnesemia is a frequent finding, affecting 10–45% of patients [123]. This may result from reduced Mg absorption in the renal tubules, as suggested by hypermagnesuria. Additionally, Mg may bind to free fatty acids, which could further contribute to the observed low serum Mg levels in these patients [124].

First-line treatment for hypomagnesemia is based on Mg supplementation, which may be administered orally or intravenously depending on the severity of the condition and has been shown to be efficient in normalizing serum Mg and Ca levels in clinical cases [125]. Nevertheless, in recent years, SGLT2 inhibitors have emerged as a promising treatment, with particular interest in refractory cases.

Numerous mechanisms may explain the effects of SGLT2 inhibitors on Mg homeostasis; however, the precise mechanisms remain unknown. At the renal level, the effects of SGLT2 inhibitors are observed in the ascending loop of Henle, where the increased sodium influx caused by these compounds activates the sodium/potassium pump, consequently altering the membrane potential gradient, ultimately leading to increased paracellular Mg reabsorption [126]. In recent years, it has been demonstrated that TRPM6 expression in the distal convoluted tubule is responsible for ~10% of the final adjustment of Mg reabsorption [127]. In this respect, studies in animals have shown that SGLT2 inhibition increases TRPM6 expression and activity without apparently affecting NKCC2 activity [128,129]. Since in vitro studies in mouse distal convoluted tubule cells have shown that glucagon increases Mg uptake [130], it is plausible to hypothesize that an elevation in glucagon levels, which is a consequence of SGLT2 inhibition [131], may lead to upregulation of TRPM6 expression, resulting in increased Mg resorption. In addition to glucagon, TRPM6 expression and activity may also be modulated by EGF [132], uromodulin or sodium/chloride pump activation [133,134], reduced oxidative stress [135], and urinary flow [136]. Notably, considering that EGF signaling triggers renal anabolic mechanisms and the inverse association of urinary EGF levels with CKD progression [107,137], this may partly explain the protective role of SGLT2 inhibitors in renal function.

The elevation in serum Mg levels was uniform across different SGLT2 inhibitors, indicating a class effect. Consequently, no distinction was observed between canagliflozin, dapagliflozin, and empagliflozin [138,139,140]. Tang et al. [138] conducted a meta-analysis to assess the effect of SGLT2 inhibitors on electrolyte levels in patients with T2DM, including 18 randomized controlled trials, and observed a significant increase in serum Mg in patients treated with SGLT2 inhibitors as compared with placebo. Similarly, in a meta-analysis by Zhang et al. [139], including 25 randomized controlled trials, the authors found an association between treatment with SGLT2 and a slight but significant increase in serum Mg (0.07 mM mean increase), with a probable more potent effect of dapagliflozin. Similarly, another study, including three clinical cases, reported the effective actions of SGLT2 inhibitors in increasing serum Mg levels, an effect that was associated with a reduction in urinary Mg excretion [141]. The effects of SGLT2 inhibitors on increasing Mg levels have also been observed in a patient receiving cisplatin as a chemotherapeutic agent who developed tubulopathy, as well as in other cases of hypomagnesemia due to the administration of other drugs, such as anticalcineurin agents [142].

Similarly, Saha et al. [143] reported a clinical case of a patient with T2DM presenting with symptomatic hypomagnesemia attributed to excessive renal Mg loss, in whom a sustained improvement in Mg levels was observed after initiation of SGLT2 inhibitors, which allowed for a reduction in the dose of daily supplements. In addition, two post-hoc analyses of randomized controlled trials enrolling 4398 and 2313 patients also showed a positive effect of dapagliflozin and canagliflozin on increasing serum Mg levels in patients with T2DM [144,145].

In 2022, Shah et al. described the first two cases demonstrating a beneficial effect on Mg levels following the initiation of SGLT2 inhibitors despite the absence of significant renal function [146]. Subsequently, they described four additional cases of drug-induced hypomagnesemia in which treatment with SGLT2 inhibitors led to a marked improvement in serum Mg levels [147]. Notably, these patients did not have T2DM, demonstrating that the effects of SGLT2 inhibitors on the elevation of serum Mg levels also occur in patients without diabetes.

Of interest, the impact of SGLT2 inhibitors on magnesemia has been observed not only in native kidneys but also in patients with KT. In a cohort of 50 KT patients treated with SGLT2 inhibitors (86% using empagliflozin), Song et al., [148] found a mean increase of 0.13 mg/dL in serum Mg after six months. Similarly, a Spanish group analyzed the efficacy and safety of SGLT2 inhibitors in KT recipients with T2DM and PTDM and found a significant increase in serum Mg levels after treatment initiation [149].

Altogether, the actions of SGLT2 inhibitors in the prevention of renal Mg wasting and the subsequent amelioration of hypomagnesemia should be particularly beneficial in patients with CKD, given the relationship between serum Mg levels and the lower risk of all-cause mortality, which has been extensively observed.

**Figure 2:**
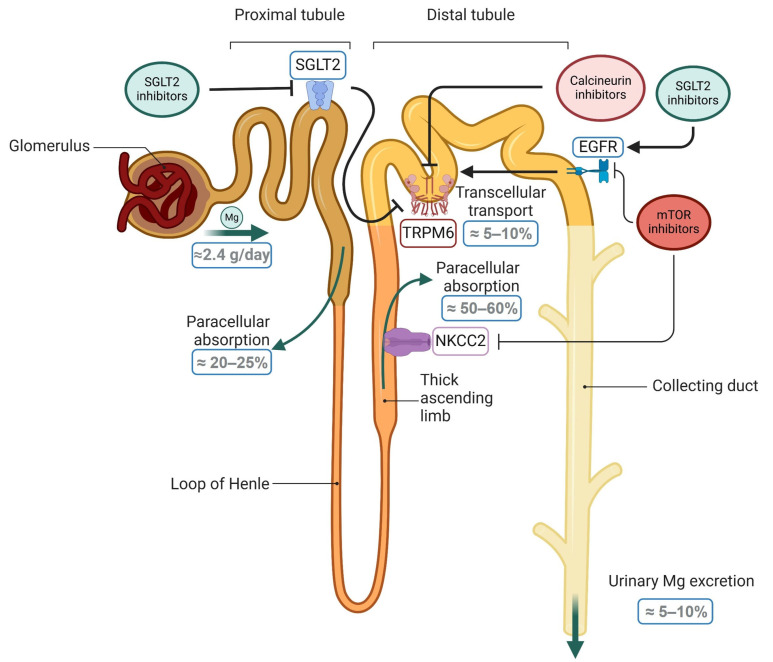
Schematic representation of the mechanisms involved in renal magnesium absorption and the potential effects of some drugs commonly used for CKD treatment. In the kidneys, most of the magnesium (Mg) is filtered by the glomerulus, which is subsequently reabsorbed paracellularly in the proximal tubule and thick ascending limb, mediated by the sodium-potassium-2chloride cotransporter 2 (NKCC2). Moreover, Mg is reabsorbed from urine into the circulation in the distal tubule in a transcellular manner through the transient receptor potential cation channel, subfamily M, member 6 (TRPM6). In this way, there are certain mechanisms that may interfere with active Mg resorption, such as glucose resorption at the proximal tubule via sodium-dependent glucose cotransporter 2 (SGLT2) or pathways involved in immunomodulatory mechanisms such as calcineurin or mammalian target of rapamycin (mTOR), which inhibit NKCC2 and epidermal growth factor (EGF) signaling, being the latter a key inductor of the TRPM6 expression. Thus, inhibitors of the SGLT2, calcineurin, and mTOR pathways, which are used in CKD patients, modulate renal Mg resorption by regulating TRPM6 expression, impacting circulating Mg levels and its associated effects on systemic mineral and bone homeostasis. Created in BioRender. Domínguez Coral, J. (2025) https://BioRender.com/r05q12e.

Figure 2 shows the interactions between treatments commonly used in CKD and KT patients, as well as the proposed crosstalk among pathways contributing to the regulation of Mg homeostasis.

## 5. Future Perspectives and Conclusions

Over the past decades, growing evidence has highlighted the key role of Mg homeostasis in regulating PTH secretion, demonstrating its effectiveness in controlling both serum PTH and phosphate levels in patients with chronic kidney disease (CKD). Moderately elevated serum Mg levels appear to be beneficial for bone health, potentially reducing the risk of fractures. However, the optimal serum Mg range remains undefined and may vary depending on the individual patient characteristics. In CKD populations, current data are inconclusive, underscoring the need for further clinical research to better understand the effects of Mg level and supplementation.

Future clinical research should prioritize patients with CKD at a higher risk of hypomagnesemia, with special attention to those with diabetes mellitus, kidney transplantation, and those receiving medications that promote Mg loss, such as proton pump inhibitors or diuretics. It is also important to highlight the need for translational studies to bridge mechanistic insights and clinical outcomes, pointing to whether Mg supplementation or other therapeutic strategies to maintain adequate serum Mg levels translate into noticeable benefits in the high-risk CKD population, such as reduction of fracture risk in patients with osteoporosis, prevention of vascular calcification in dialysis patients, improvement of metabolic outcomes during the post-transplant period, or mitigation of persistent SHPT.

In clinical practice, it seems reasonable to avoid hypomagnesemia using targeted strategies adapted to specific CKD populations. Patients with a high vascular calcification burden or uncontrolled SHPT may benefit from increasing the dialysate Mg concentration, while Mg-based phosphate binders (e.g., magnesium carbonate) should be prioritized over Ca-based alternatives to reduce the calcium load. SGLT2 inhibitors may be particularly useful in patients with hypomagnesemia who show intolerance to oral Mg formulations, particularly in KT recipients at a high risk of developing post-transplant diabetes. These approaches highlight the need for personalized Mg supplementation strategies based on the patient profile, carefully selecting the adequate formulation, and monitoring serum Mg levels to optimize safety and efficacy.

In summary, while experimental and early clinical data support the beneficial role of Mg in CKD-MBD, further well-designed studies are essential to delineate optimal therapeutic strategies and translate this knowledge into improved clinical outcomes.

## Abbreviations

The following abbreviations are used in this manuscript:

Mg	Magnesium
P	Phosphorus
Ca	Calcium
CKD	Chronic kidney disease
KT	Kidney transplant
SGLT2	Sodium-glucose co-transporter 2
TRPM7	Transient Receptor Potential Cation Channel Subfamily M Member 7
TRPM6	Transient Receptor Potential Cation Channel Subfamily M Member 6
PTH	Parathyroid hormone
FGFR1	Fibroblast Growth Factor Receptor 1
VDR	Vitamin D receptor
CaSR	Calcium-Sensing Receptor
P2X7	P2X purinoceptor 7
NMDA	N-methyl-D-aspartate
Magt1	Magnesium Transporter 1
SHPT	Secondary hyperparathyroidism
HD	Hemodialysis
TNFα	Tumor necrosis factor alpha
IL	Interleukin
BALP	bone alkaline phosphatase
TRAP5b	Tartrate-Resistant Acid Phosphatase 5 b
CPPs	Calciprotein particles
T2DM	Type 2 diabetes mellitus
mTOR	Mammalian target of rapamycin
CNIs	Calcineurin inhibitors
NKCC2	sodium-potassium-2chloride cotransporter 2
EGF	Epidermal growth factor
PTDM	Post-transplantation diabetes mellitus
PPIs	Proton pump inhibitors
